# Efficacy and safety evaluation of SGLT2i on blood pressure control in patients with type 2 diabetes and hypertension: a new meta-analysis

**DOI:** 10.1186/s13098-023-01092-z

**Published:** 2023-06-07

**Authors:** Qin Zhang, Siyuan Zhou, Lijun Liu

**Affiliations:** 1 Guang’anmen Hospital, China Academy of Chinese Medical Sciences, Beijing, 100053 China; 2 South Hospital Area of the Fifth Medical Center of the PLA General Hospital, Beijing, 100053 China

**Keywords:** Type 2 diabetes and hypertension, SGLT2i, Meta-analysis

## Abstract

**Background:**

The clinical efficacy and safety of SGLT2i in treating patients with type 2 diabetes mellitus and hypertension lack immense data support.

**Objective:**

To systematically evaluate the clinical efficacy and safety of SGLT2i in patients with type 2 diabetes mellitus and hypertension by collecting the previously published randomized controlled trials on SGLT2i to provide data support for SGLT2i as an adjuvant in the first-line antihypertensive regimen in patients with type 2 diabetes mellitus and hypertension.

**Methods:**

Randomized controlled trials comparing SGLT2i with a placebo in treating type 2 diabetes with hypertension were screened strictly according to inclusion and exclusion criteria. The primary efficacy endpoints included 24H systolic blood pressure, 24H diastolic blood pressure, office systolic blood pressure, and office diastolic blood pressure. The secondary efficacy endpoints included HbA1c. The safety indicators were hypoglycemia, urinary tract infection, genital infection, and renal impairment.MD was the combined effect size for continuous variables, and RR was the combined effect size for dichotomous variables.

**Results:**

10 RCTs with 9913 participants (6293 in the SGLT2i group and 3620 in the control group) were included for analysis.SGLT2i is effective in reducing blood pressure compared with placebo in patients with type 2 diabetes and hypertension, 24HSBP (− 5.06 mmHg, 95% CI [− 7.10, − 3.01], t = − 6.19, P < 0.05), 24HDBP (− 2.39 mmHg, 95% CI [− 4.11, − 0.67], t = − 4.15, P = 0.004), Office SBP (− 4.53 mmHg, 95% CI [− 5.66, − 3.40], t = − 9.50, P < 0.05), Office DBP (− 2.12 mmHg, 95% CI [− 3.42, − 0.82], t = − 4.88, P = 0.001). HbA1c decreased significantly (− 0.57%, 95% CI [− 0.60, − 0.54], z = 37.02, p < 0.01). SGLT2i did not increase hypoglycemia compared to placebo (RR = 1.22, 95% CI [0.916, 1.621], z = 1.36 p = 0.174), urinary tract infection (RR = 1.56, 95% CI [0.96, 2.52], z = 1.79 p = 0.073), risk of renal injury (RR = 0.78, 95% CI [0.54, 1.13], Z = 1.31, P = 0.19), but the risk of genital tract infection increased by 2.32 times (RR = 2.32, 95% CI [1.57, 3.42], Z = 4.23, P = 0.00).

**Conclusion:**

SGLT2i can effectively control blood pressure and blood glucose and generally has high safety. For patients with type 2 diabetes mellitus and hypertension with a low risk of genital infection, SGLT2i should be considered as an adjuvant drug for a first-line antihypertensive regimen.

## Introduction

Hypertension and diabetes mellitus are independent risk factors for cardiovascular disease [1]. The risk of cardiovascular disease in type 2 diabetes is three times that of non-diabetic patients. In China, the prevalence of hypertension is nearly 90% among the elderly aged ≥ 80 years old, which is the primary risk factor for stroke, myocardial infarction, and even cardiovascular death. Patients with type 2 diabetes often have hypertension, and the incidence of hypertension in patients with diabetes is twice that in patients without diabetes [2]. Although diabetes mellitus and hypertension are increasing yearly, the two diseases’ awareness, treatment, and control rates are still low in different countries. Blood glucose and blood pressure levels are continuously, independently, and positively related to cardiovascular risk [3]. The risk of cardiovascular complications in patients with type 2 diabetes and hypertension is 2 to 4 times higher than in patients with common diabetes mellitus or hypertension, and the risk of all-cause mortality and cardiovascular events is 1.7 times and 1.5 times higher, respectively, than in patients with normal blood pressure [4]. Therefore, strict control of blood sugar and blood pressure can effectively reduce cardiovascular and cerebrovascular complications.

According to the latest China guidelines, the blood pressure reduction target for patients with type 2 diabetes mellitus and hypertension is < 130/80 mmHg [5]. This requires a combination of antidiabetic and antihypertensive drugs to minimize the risk of later cardiovascular disease and avoid the increased morbidity and mortality associated with drug abuse. Traditional antihypertensive drugs are not effective in blood glucose management. The high incidence of hypertension and cardiovascular risk in type 2 diabetes mellitus urges us to find a safe and effective antihypertensive drug. As a new antidiabetic drug, the antihypertensive effect of sodium-glucose co-transporter 2 (SGLT2i) inhibitors has been confirmed [6, 7]. Meta-analyses have shown that SGLT2i significantly reduces systolic and diastolic blood pressure in patients with diabetes, with renal and cardiovascular protection [8]. However, there is still a lack of big data to support how to guide the use of such drugs in patients with type 2 diabetes mellitus and hypertension, as well as the discussion on the safety and effectiveness of SGLT2i as an add-on to first-line antihypertensive drugs in patients with diabetes mellitus and hypertension.

In summary, finding the ideal drug to achieve both blood pressure and glycemic goals, avoid the side effects of multiple drugs, and achieve optimal efficacy is daunting. The use of SGLT2i could be beneficial to achieve this goal, and this study was conducted with this aim in mind.

## Methods

### Literature screening methods and data sources

This study was conducted strictly with PRISMA reporting principles and registered in the International Prospective Registry of Systematic Reviews (PROSPERO: CRD42023400975). The Chinese and English databases of Pubmed, Embase, Cochrane Library, web of science, SinoMed, CNKI, VIP, and Wanfang were searched with diabetes, hypertension, and SGLT2i as the subject words in the mode of “subject words + free words” from the establishment of the database to December 2022. Further full-text assessments are obtained for relevant citations that appear in the literature.

### Literature inclusion criteria

(1) Comparative analysis of the efficacy of SGLT2i (any dose) and control group (placebo or no SGLT2i) in patients with type 2 diabetes mellitus and hypertension, efficacy measures including blood pressure (24HSBP/DBP or average office SBP/DBP). (2) The study type is a randomized controlled intervention study. (3) The shortest follow-up time was 4 weeks. (4) The research data are accurate and complete, and the research subjects are at least 18 years old and above, regardless of region, country, nationality, and language.

### Literature exclusion criteria

(1) Non-randomized controlled trials. (2) Conference documents and other data are incomplete. (3) The same experimental data were published repeatedly. (4) The sample size is less than 60 cases. (5) The loss to follow-up rate during follow-up is more than 20%.

### Literature quality evaluation

The literature quality evaluation scale provided by Cochrane Collaboration was used to evaluate the quality of the included literature, mainly including the generation of random sequence, concealment of the allocation scheme, double-blind of investigators and subjects, blind evaluation of study conclusion, the integrity of outcome data, selective reporting of study results and bias from other sources.

### Data extraction and outcome measures

From literature screening to data extraction, two researchers of this specialty independently completed the study according to the inclusion and exclusion criteria. For the literature with disagreement, the third investigator of this specialty will participate in the adjudication. The literature was preliminarily screened by titles and abstracts, and the literature irrelevant to the study content and study type was excluded. The integrity and usability of the study data were further examined by reading the full text. The extraction contents include (1) the authors, years, basic information of subjects, disease severity, sample size, intervention measures, course of treatment, outcome indicators, etc. of the included literature; (2) key factors of document quality evaluation; (3) specific values of outcome indicators.

### Outcome measures

Primary outcome measures included 24H systolic and diastolic blood pressure, mean office systolic and diastolic blood pressure; Secondary outcome measures included HbA1c. Safety indicators included hypoglycemia, urinary tract infection, genital tract infection, and renal injury.

### Data synthesis and statistical analysis

Continuous variables were expressed as mean ± SD, with a mean difference (MD) as the final effect size and a 95% confidence interval (95% CI). Categorical variables were expressed as frequencies and percentages, and the final effect size was assessed as risk ratio (RR) and 95% confidence interval (95% CI). All p-values were 2-tailed, and 0.05 was considered statistically significant. I^2^ is an indicator for evaluating heterogeneity, and I^2^ < 25% is considered as slight heterogeneity; I^2^ < 50%, moderate heterogeneity; I^2^ > 50%, highly heterogeneous. The random effect model is preferred for meta-analysis, and the fixed effect model is selected for the results with small I^2^. The preferential random effect model is based on the interference between different studies, especially the factors such as subject age, disease severity, and primary treatment regimen. Further sensitivity analysis and star plot were performed to analyze the sources of heterogeneity. All analyses were performed using software version Stata14.0.

## Result

### Literature characteristics of included studies

**Fig. 1:**
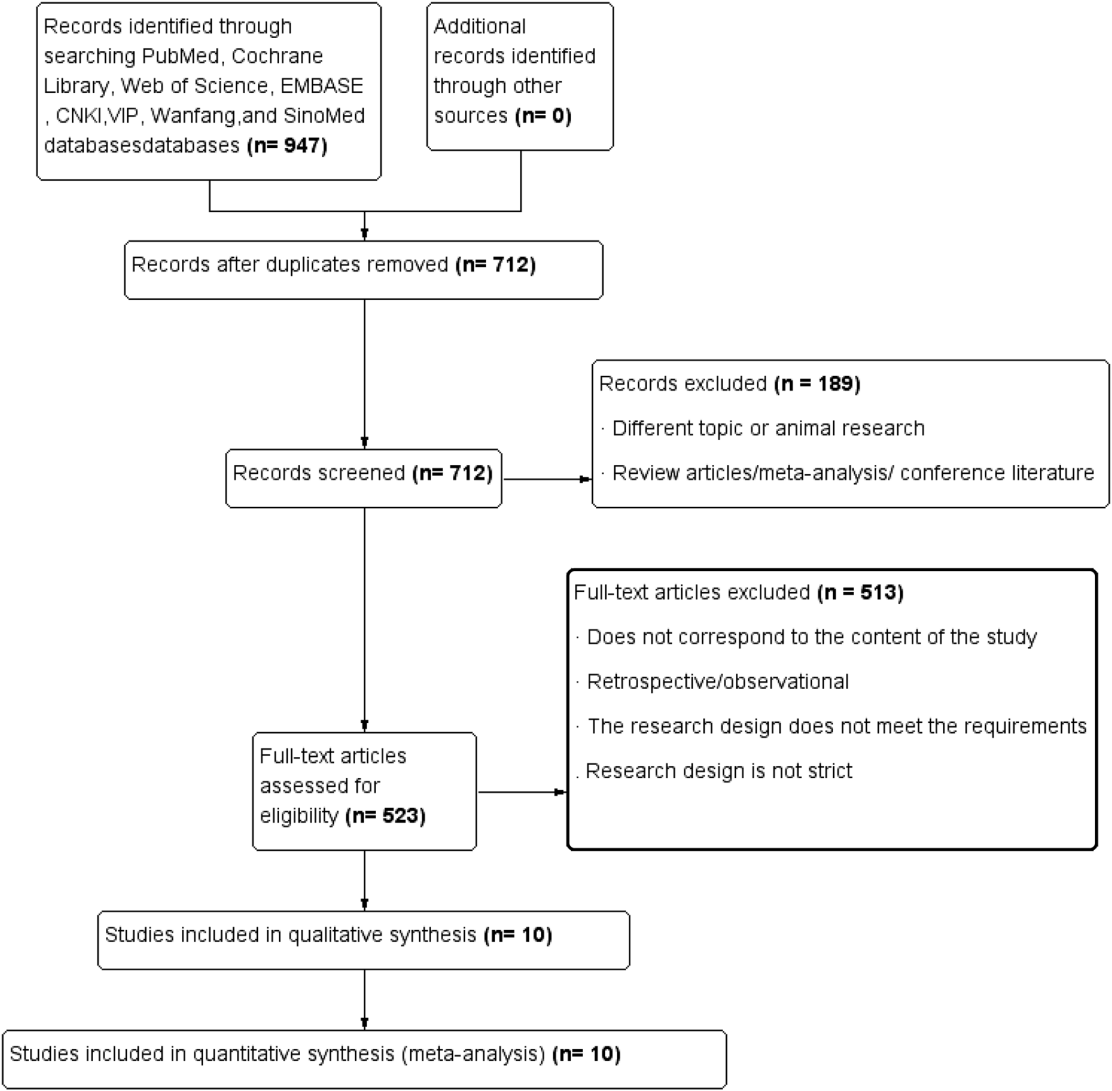
Flow chart of literature screening

A total of 947 pieces of literature were retrieved, and ten pieces of literature [2, 9–17] were finally included in the analysis (literature 20 set subgroups, respectively refractory hypertension and non-refractory hypertension), with a total of 9913 participants (including 6293 in SGLT2i group and 3620 in the control group). The detailed screening process is shown in Fig. 1. Table 1 reports the basic information of the included articles.

**Table 1:** Basic information of included studies

Study (year)	Intervention measures	Dosage	Outcomes	Basic treatment plan	Course of treatment
Keith C2019	Empagliflozin/placebo	10–25 mg	HcA1c, 24H-SBP/DBP, office SBP/DBP	Basic antihypertensive and hypoglycemic program	12/24 week
Kazuomi 2019	Empagliflozin/placebo	10 mg	24H SBP/DBP, HbA1, body weight	Basic hypoglycemic and antihypertensive drugs, including ARB	12 week
Michael 2015	Dapagliflozin/placebo	10 mg	24HSBP, office SBP, HbA1c, body weight	Basic hypoglycemic and antihypertensive drugs, including ARB/ACEI	12 week
Tikkanen 2015	Empagliflozin/placebo	10/25 mg	24H SBP/DBP, office SBP/DBP, 55.1/54.8HbA1, body weight	Basic antihypertensive and hypoglycemic program	12 week
Heerspink 2016	Dapagliflozin/placebo	10 mg	Office SBP/DBP, HbA1, body weight	Basic hypoglycemic and antihypertensive drugs, including ARB/ACEI	12 week
Ferreira 2020	Empagliflozin/placebo	10/25 mg	Office SBP, HbA1, body weight	Basic hypoglycemic and antihypertensive drugs, including diuretics	14 week
LanCheng 2021	Empagliflozin/placebo	25 mg	24HSBP/DBP, office SBP/DBP	Basic hypoglycemic and antihypertensive drugs	12 week
Amin 2011	Empagliflozin/placebo	25 mg	24HSBP/DBP	Stop the basic hypoglycemic and antihypertensive regimen 3 weeks before randomization	4 week
Raymond 2015	Canagliflozin/placebo	100/300 mg	24HSBP/DBP	Basic hypoglycemic and antihypertensive drugs, including ARB/ACEI	6 week
Weber 2015	Dapagliflozin/placebo	10 mg	Office SBP/DBP, HbA1c, 24H SBP	Basic hypoglycemic and antihypertensive drugs, including ARB/ACEI	12 week

### Baseline characteristics of included cohorts

Table 2 lists the subjects’ baseline data and basic information. The study population of the SGLT2i group and the control group are comparable in blood pressure, blood glucose control level, disease duration, disease severity grading, etc.

**Table 2:** Baseline data of the experimental population included in the study

Study (year)	Age, y	Male, %	Number of Patients	diabetes duration (year)	BMI (kg/m^2^)	SBP (mmHg)	HbA1C (%)	Study type
Keith C2019	57.2/56.5	50/55.1	72/78	9.3/9.3	35.12/36.04	–	–	RCT
Kazuomi 2019	69.3/70.9	52.4/52.9	63/68	9.6/10.6	26/26.1	–	6.6/6.6	RCT
MichaelA 2015	56.2/55.6	55/59.3	311/302	7.6/8.2	–	149.5/149.8	8.0/8.1	RCT
Tikkanen 2015	60.3/60.6/59.9	62/62/56.5	271/276/276	–	32.4/32.4/33	131.7/131.3/131.2	7.9/7.87/7.92	RCT
Heerspink 2016	55.1/54.8	69.8/57.5	189/167	8.3/8.6	31.3/31.6	151.4/151.9	8.1/8.1	RCT
Ferreira1 2020	–	–	516/1063	–	–	–	–	RCT
Ferreira2 2020	–	–	1817/3624	–	–	–	–	RCT
LanCheng 2021	71.7/71.2	59.7/64.5	62/62	–	25.8/25.5	150.8/153.0	7.7/7.7	RCT
Amin 2011	–	–	39/39	–	–	–	–	RCT
Raymond 2015	58.3/57.8/59.6	55.4/59.6/58.9	56/57/56	–	34.1/33.0/32.9	139.6/136.5/136.7	8.0/8.1/8.2	RCT
Weber 2015	57/56	58/52	224/225	7.3/7.7	–	151.3/151.0	8.0/8.1	RCT

All values are reported as Control or Placebo/SGLT2i. Data for mean value for the whole population. The missing data in the table is due to the different reporting methods of the original test data. The baseline data of the experimental group and the control group were comparable

### Literature quality assessment

**Fig. 2:**
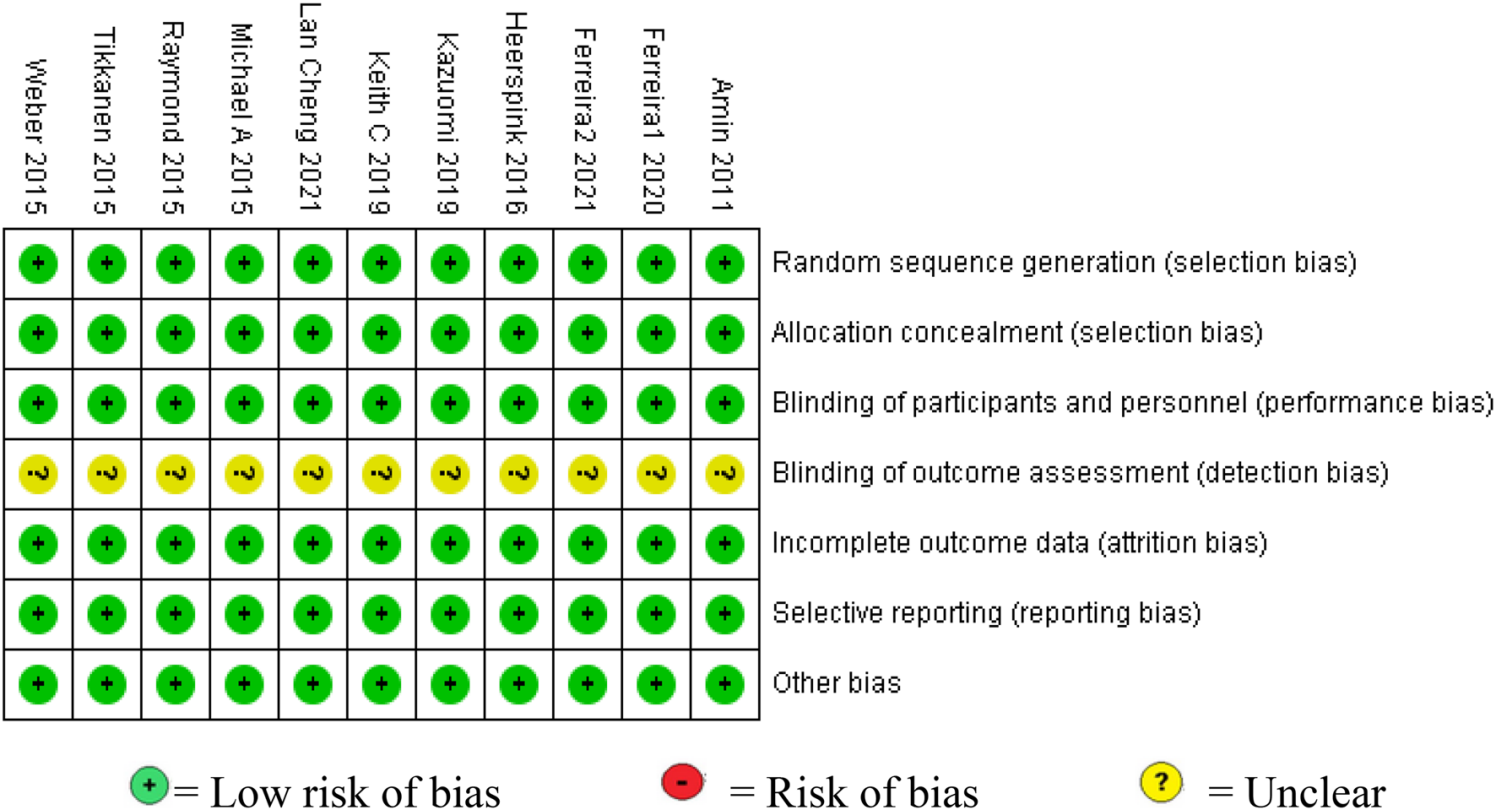
Literature quality assessment

The Cochrane Collaboration’s risk of bias tool was used (Fig. 2).

### Efficacy evaluation and outcome

#### 24H systolic blood pressure

**Fig. 3:**
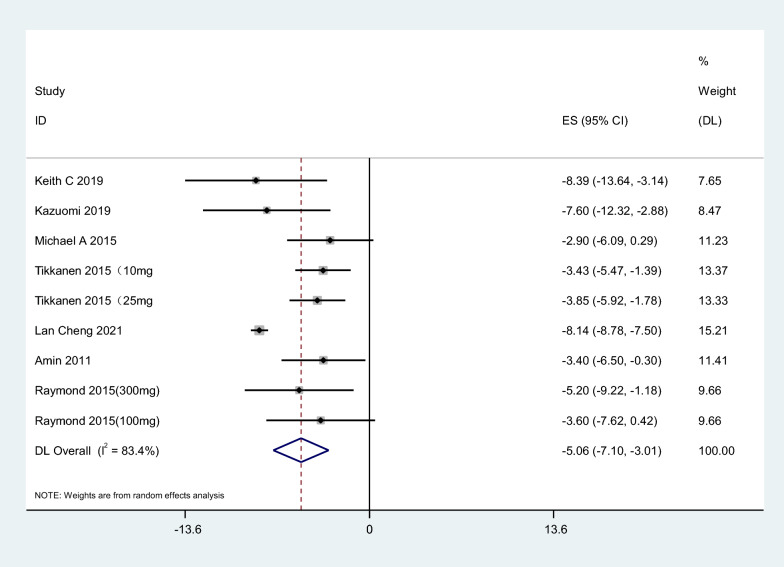
Effect of SGLT2i on 24-h systolic blood pressure

Seven articles were included in the study. The results showed that SGLT2i could reduce 24HSBP by 5.06 mmHg compared with placebo, 95% CI [− 7.10, − 3.01], t = − 6.19, P < 0.05, and the difference was statistically significant. The heterogeneity test showed that I^2^ = 83.4%, P < 0.1, showing strong heterogeneity among literature (Fig. 3). Considering the differences in the types, doses, and courses of SGLT2i in the included studies, which may cause heterogeneity, further subgroup analysis was performed, and the results were consistent (Table 3).

**Table 3:** Subgroup analysis of the effect of SGLT2i on blood pressure

Subgroup	MD	95% CI	I^2^ (%)	P
24H SBP				
SGLT2i				
Empagliflozin	− 7.30	− 7.86, − 6.73	86.6	0.000
Dapagliflozin	− 2.9	− 6.09, 0.29	–	0.075
Canagliflozin	− 4.4	− 7.25, − 1.56	0.0	0.002
Dose				
Large dose	− 7.41	− 7.99, − 6.83	84.3	0.000
Small dose	− 4.00	− 5.70, − 2.30	22.3	0.000
Course of treatment				
> 6 week	− 7.29	− 7.85, − 6.72	87.1	0.000
≤ 6 week	− 3.94	− 6.04, − 1.85	0.0	0.000
24H DBP				
SGLT2i				
Empagliflozin	− 4.90	− 5.14, − 4.67	94.9	0.000
Dapagliflozin	− 0.7	− 2.87, − 1.47	–	0.52
Canagliflozin	− 2.45	− 4.12, − 0.78	0.0	0.004
Dose				
Large dose	− 4.99	− 5.23, − 4.75	0	0.000
Small dose	− 2.11	− 3.04, − 1.17	94.0	0.000
Course of treatment				
> 6 week	− 4.90	− 5.13, − 4.66	95.1	0.000
≤ 6 week	− 2.07	− 3.36, − 0.77	0.0	0.002
Office systolic pressure				
SGLT2i				
Empagliflozin	− 6.03	− 6.36, − 5.71	66.9	0.000
Dapagliflozin	− 3.55	− 4.98, − 2.13	0.0	0.000
Dose				
Large dose	− 5.97	− 6.29, − 5.65	72.4	0.000
Small dose	− 4.1	− 5.88, − 2.32	0.0	0.000
Course of treatment				
≤ 12 week	− 6.06	− 6.39, − 5.72	68.3	0.000
> 12 week	− 4.13	− 5.29, − 2.98	0.0	0.000
Office diastolic pressure				
SGLT2i				
Empagliflozin	− 3.38	− 3.58, − 3.18	97.0	0.000
Dapagliflozin	− 0.99	− 2.10, − 0.12	0.0	0.082
Dose				
Large dose	− 3.40	− 3.60, − 3.20	97.1	0.000
Small dose	− 1.94	− 2.71, − 1.17	0.0	0.000
Course of treatment				
≤ 12 week	− 4.09	− 4.32, − 3.86	92.7	0.000
> 12 week	− 1.44	− 1.80, − 1.08	0.0	0.000

#### 24H diastolic blood pressure

**Fig. 4:**
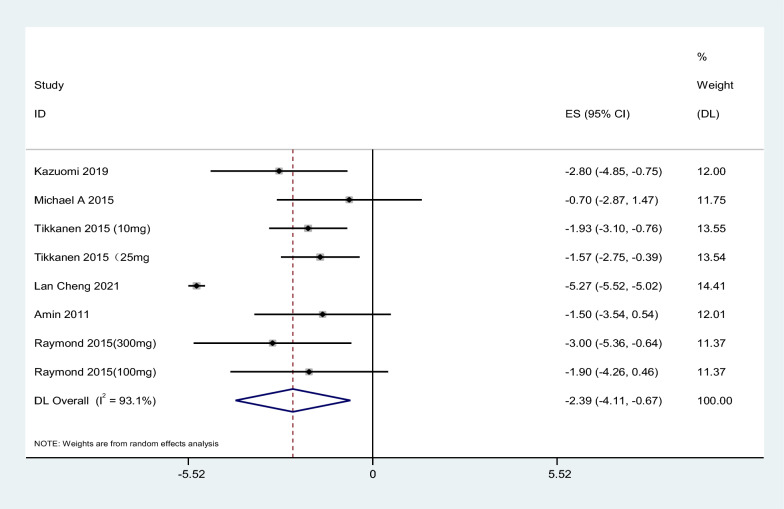
Effect of SGLT2i on 24H diastolic blood pressure

A total of 6 kinds of literature were included in the study. The results showed that SGLT2i could reduce 24HDBP by 2.39 mmHg compared with placebo, 95% CI [− 4.11, − 0.67], t = − 4.15, P = 0.004, and the difference was statistically significant. The heterogeneity test showed that I^2^ = 93.1%, P < 0.1, showing firm heterogeneity among literature, as shown in Fig. 4. Further subgroup analysis of SGLT2i type, dose, and treatment course showed consistent results (Table 3).

#### Office systolic blood pressure

**Fig. 5:**
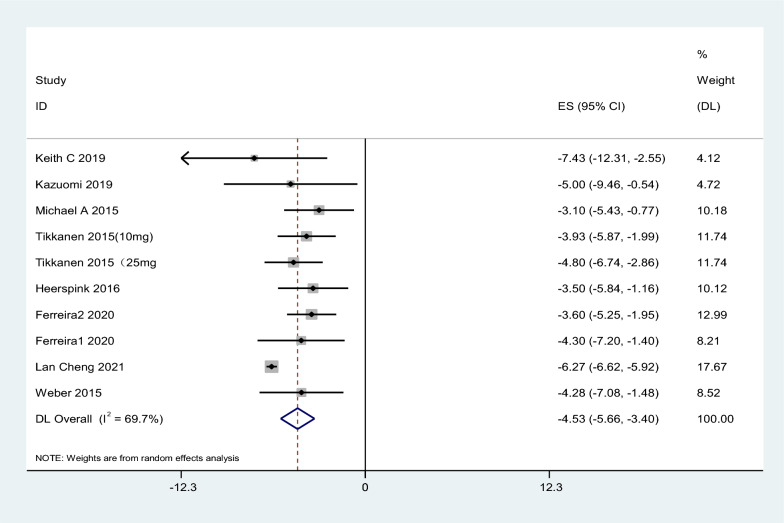
Effect of SGLT2i on office systolic blood pressure

A total of 8 works of literature were included in the study. The results showed that SGLT2i could reduce office SBP by 4.53 mmHg compared with placebo, 95% CI [− 5.66, − 3.40], t = − 9.50, P < 0.05, and the difference was statistically significant. The heterogeneity test showed that I^2^ = 69.7%, P < 0.1, showing substantial heterogeneity among literature, as shown in Fig. 5. Further subgroup analysis of SGLT2i type, dose, and treatment course showed consistent results (Table 3).

#### Office diastolic blood pressure

**Fig. 6:**
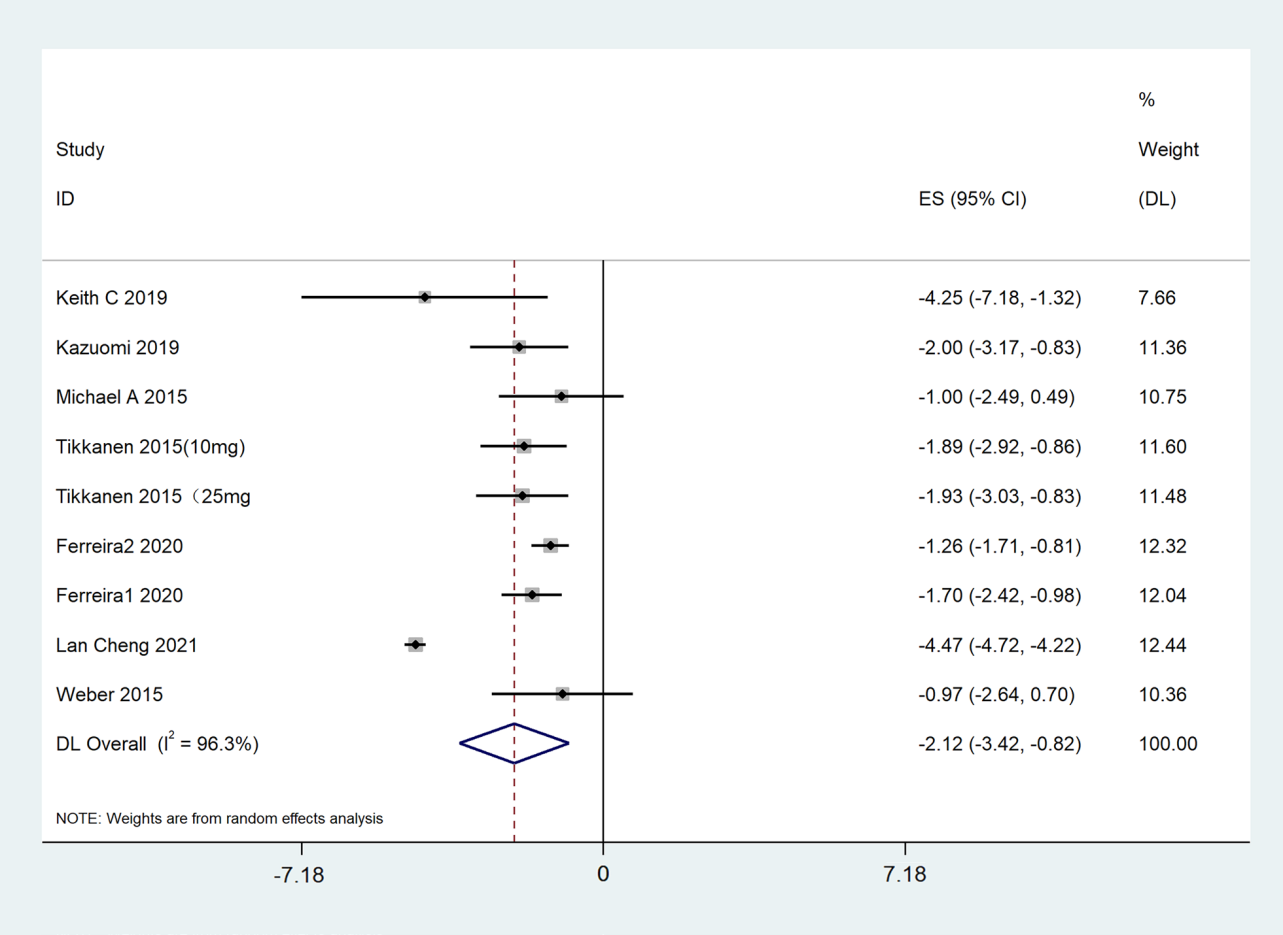
Effect of SGLT2i on office diastolic blood pressure

A total of 7 pieces of literature were included in the study. The results showed that SGLT2i reduced office DBP by 2.12 mmHg compared with placebo, 95% CI [− 3.42, − 0.82], t = − 4.88, P = 0.001, and the difference was statistically significant. The heterogeneity test showed that I^2^ = 96.3%, showing substantial heterogeneity among literature, as shown in Fig. 6. Further subgroup analysis of SGLT2i type, dose, and treatment course showed consistent results (Table 3).

#### HbA1c

**Fig. 7:**
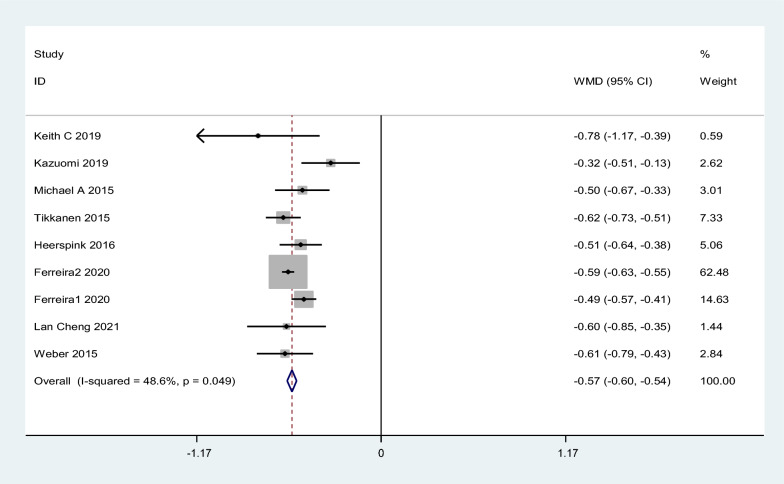
Effect of SGLT2i on HbA1c

A total of 8 works of literature were included in the study. The results showed that SGLT2i could reduce HbA1c by 0.57% compared with placebo, with 95% CI [− 0.60, − 0.54], z = 37.02, p < 0.1, and the difference was statistically significant. Heterogeneity test I^2^ = 48.6%, Fig. 7

#### Sensitivity analysis

The sensitivity analysis of the effect of SGLT2i on blood pressure showed this literature [2] showed high sensitivity. The heterogeneity of the effect of SGLT2i on 24HSBP after excluding this literature was I^2^ = 2.8%, P > 0.1, combined effect size MD = − 4.03, 95% CI [5.10, − 2.97], z = 7.41 p = 0.000. After the literature [2] was excluded, the heterogeneity of the effect of SGLT2i on office SBP was I^2^ = 0%, P > 0.1, combined effect size MD = − 4.03, 95% CI [− 4.82, − 3.24] z = 10.02 p = 0.000. After excluding the literature [2], the heterogeneity of the effect of SGLT2i on 24HDBP, I^2^ = 0%, P > 0.1, combined effect size MD = − 1.84, 95% CI [− 2.47, − 1.21] z = 5.7 p = 0.000. After the literature [2] was excluded, the heterogeneity of the effect of SGLT2i on office DBP was I^2^ = 6.2%, P > 0.1, combined effect size MD = − 1.52, 95% CI [− 1.83, − 1.21] z = 9.59 p = 0.000.

### Safety evaluation

#### Hypoglycemia

**Fig. 8:**
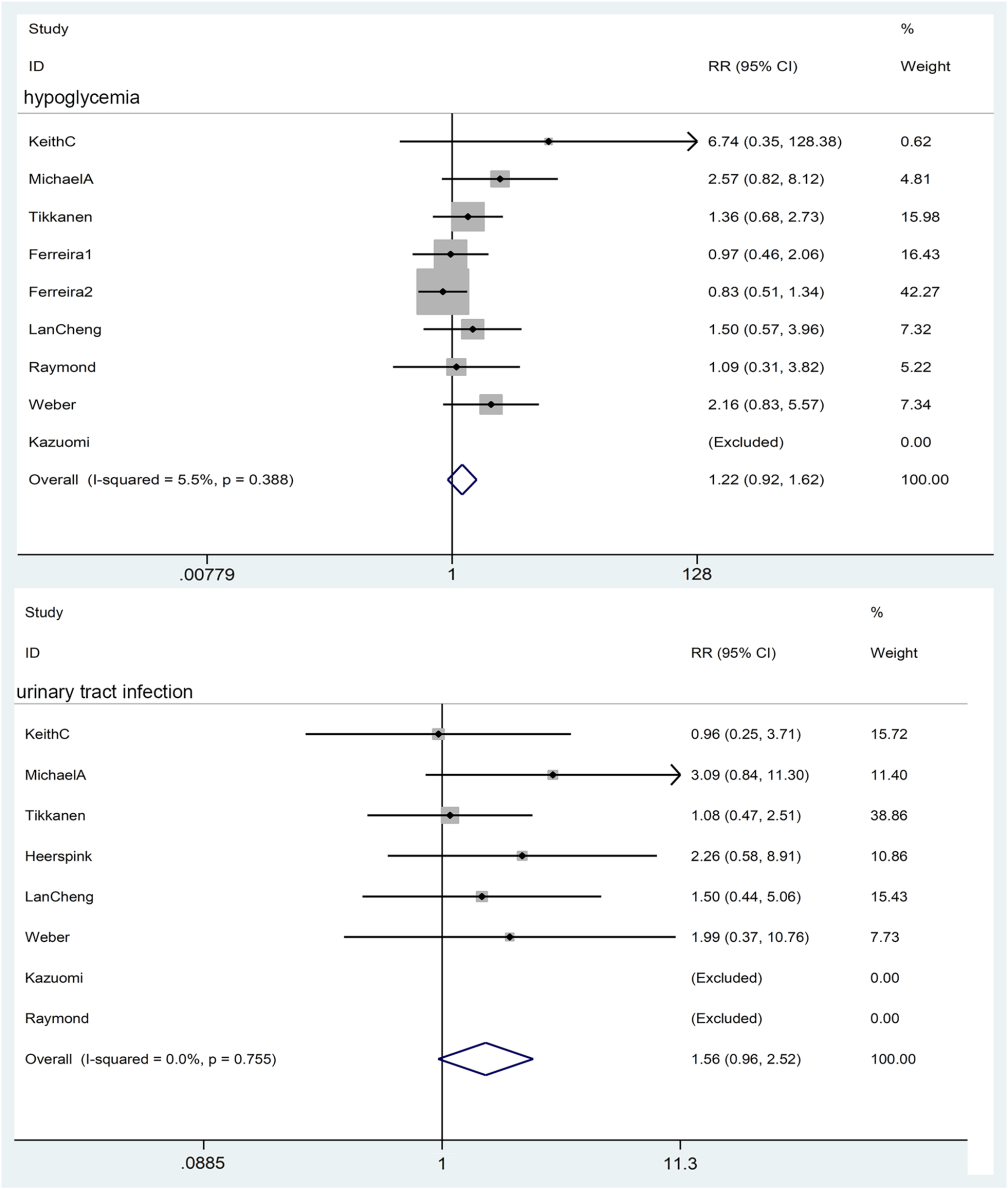
Effect of SGLT2i on hypoglycemia and urinary tract infection

A total of 8 pieces of literature were included in the study, and the heterogeneity test showed I^2^ = 5.5%, P = 0.39, indicating no heterogeneity, RR = 1.22, 95% CI [0.916, 1.621], z = 1.36 p = 0.174. The results suggest that SGLT2i does not increase the risk of hypoglycemia compared with placebo (Fig. 8).

#### Urinary tract infection

A total of 8 studies on urinary tract infection were included. The heterogeneity test showed I^2^ = 0%, P = 0.76, indicating no heterogeneity, RR = 1.56, 95% CI [0.96, 2.52], meta-analysis result z = 1.79, p = 0.073, indicating SGLT2i did not increase the risk of urinary tract infection compared with placebo (Fig. 8).

#### Genital tract infection

**Fig. 9:**
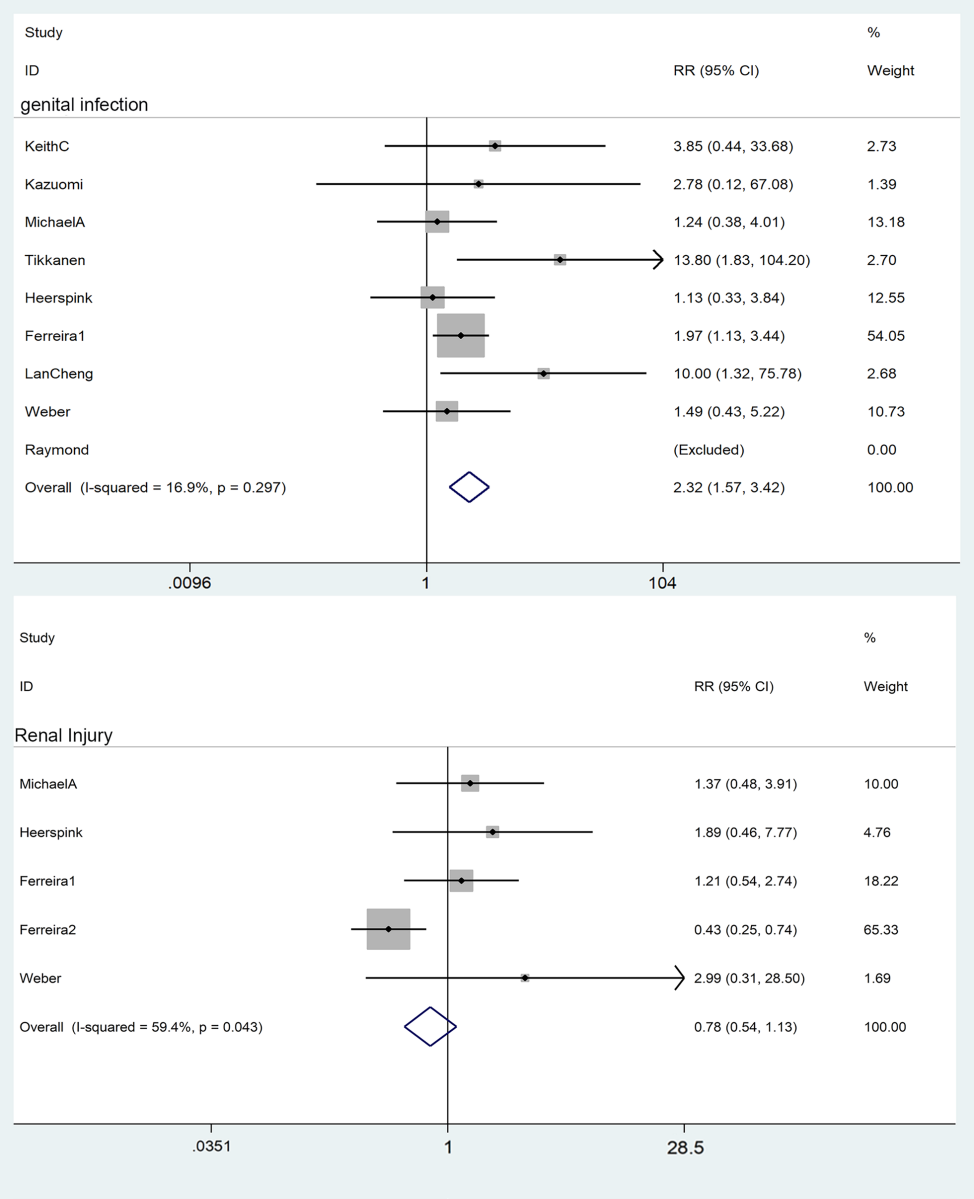
Effect of SGLT2i on genital infection and renal injury

A total of 9 works of literature were included in the study. The heterogeneity test showed that I^2^ = 49.9%, P = 0.00, indicating moderate heterogeneity. A star diagram was further drawn to judge heterogeneity, and the source of heterogeneity was analyzed by sensitivity. Ferreira [9] showed high sensitivity. After excluding this literature [9]: I^2^ = 16.9%, RR = 2.32, 95% CI [1.57, 3.42], z = 4.23, p = 0.00, suggesting a 2.32-fold increased risk of genital infections for SGLT2i compared with placebo (Fig. 9).

#### Renal injury

A total of 7 pieces of literature compared the incidence of acute kidney injury, among which, no renal injury occurred in three literature [13, 16, 17] in both the experimental and the control groups. Therefore, four pieces of literature were finally included in the meta-analysis. I^2^ = 59.4%, RR = 0.78, 95% CI [0.54, 1.13], Z = 1.31, P = 0.19, indicating that SGLT2i does not cause renal injury compared with placebo (Fig. 9).

## Discussion

The global prevalence of diabetes has risen sharply. According to statistics, in the past 40 years, 34.2 million people in the United States have diabetes, 95% of whom are type 2 diabetes. The risk of cardiovascular disease in adults with diabetes accounts for 10–12% of all vascular deaths compared with adults without diabetes [18]. Hypertension is a common disease coexisting with diabetes mellitus and a definite risk factor for Atherosclerotic cardiovascular disease (ASCVD). Two-thirds of patients with diabetes have hypertension, and the risk of cardiovascular disease in patients with diabetes with hypertension is four times higher than that in normotensive patients with diabetes [19]. Patients with type 2 diabetes mellitus and hypertension have a significantly higher risk of complications such as myocardial infarction, stroke, and chronic renal failure (CKD) [20]. A substantial benefit is seen when multiple cardiovascular risk factors are resolved simultaneously. Therefore, active control of blood pressure is essential for people with type 2 diabetes.

WHO recommends that antihypertensive drugs be started when systolic blood pressure is 130–139 mmHg in patients diagnosed with hypertension, concomitant cardiovascular disease, or high cardiovascular risk (such as diabetes mellitus or chronic kidney disease).In the initial treatment, any two of angiotensin-converting enzyme inhibitors (ACEIs)/angiotensin-receptor blockers (ARBs), diuretics (thiazide or thiazide-like) and long-acting dihydropyridine calcium channel blockers (CCBs) can be selected for combination therapy. Compared with CCBs and β-blockers, ACEIs/ARBs can better reduce the risks of MACE, heart failure, cardiovascular death, and all-cause death and are the first drugs of choice for treating hypertension in patients with diabetes mellitus and coronary artery disease [21]. For patients with diabetes and hypertension, blood pressure targets should be individualized through a shared decision-making process that addresses cardiovascular risk, potential adverse effects of antihypertensive medications, and patient preferences [21]. Maintaining blood pressure and blood glucose at an ideal level is complicated, and multi-drug combination therapy is required. Therefore, excessive treatment should be avoided when formulating a treatment regimen to reduce the occurrence of adverse drug events [22]. Currently, the choice of therapeutic drugs for patients with type 2 diabetes mellitus complicated with hypertension still lacks extensive data support at home and abroad.

As a new oral antidiabetic drug, SGLT2i reduces renal reabsorption of glucose and sodium by inhibiting sodium-glucose co-transporter 2, thus exerting hypoglycemic and antihypertensive effects [23, 24]. Recently, Sha Chen proposed that myocardial cells will start intracellular plasmalemma sodium loaders when stimulated by hypoxia, oxidative stress, validation, acidosis, etc. The protective effect of SGLT2i on the heart and kidney benefits from their inhibition of sodium loaders in the plasma membrane (NHE-1, Nav1.5, SGLT) affecting intracellular sodium-homeostasis (the sodium-interactome), thus reducing corresponding damage [25]. Compared with previous antidiabetic drugs, such as sulfonylureas (sulfonylureas), biguanides, and thiazolidinediones (TZDs), SGLT2i can reduce the occurrence of adverse drug events such as cardiovascular, liver function and renal function injury [26, 27]. The CCS/CHFS heart failure guidelines recommend SGLT2i for preventing or treating heart failure in patients with or without type 2 diabetes [28]. Clinical trials have demonstrated that SGLT2i drugs such as empagliflozin, canagliflozin, and GLP-1 receptor agonists reduce the risk of cardiovascular adverse events, and Dapagliflozin reduces the risk of cardiovascular death or hospitalization for heart failure [5, 29]. Dapagliflozin effectively reduces albuminuria after renin-angiotensin system blockade therapy in patients with T2DM and hypertension, improves glycemic control, reduces systolic blood pressure, and promotes uric acid excretion, thus reducing renal and cardiovascular risks [15]. Empagliflozin reduced major cardiovascular deaths regardless of treatment-resistant hypertension status. Arterial stiffness is an established risk factor for cardiovascular events and mortality. The underlying mechanism of blood pressure reduction by SGLT2i may be related to the improvements in endothelial function and arterial stiffness associated with empagliflozin [2]. SGLT2i induced clinically relevant reductions in systolic blood pressure and HbA1c, a dual effect that should be considered in patients with hypertension and T2D, as demonstrated in this study.

Systolic blood pressure is more clinically significant than diastolic blood pressure in patients with hypertension. The former is a more robust indicator of the risk of cardiovascular disease, which has led to an increasing emphasis on systolic blood pressure control in recent guidelines. This study preliminarily confirmed that SGLT2i can reduce 24-h systolic blood pressure and office systolic blood pressure in patients with type 2 diabetes and hypertension. SGLT2 reduced 24H systolic blood pressure by 5.06 mmHg and office systolic blood pressure by 4.53 mmHg, on average, compared with the equivalent dose of placebo. For 24H and mean office diastolic blood pressure, SGLT2i also showed a certain antihypertensive effect. The antihypertensive efficacy of SGLT2i is partly influenced by choice of first-line antihypertensive agents. Because SGLT2i lowers blood pressure by increasing urine volume, Dapagliflozin’s blood pressure lowering properties are especially beneficial in patients who are already taking beta-blockers or calcium channel blockers, and the addition of Dapagliflozin may result in slightly more tremendous blood pressure lowering than in patients who are already taking thiazide diuretics [12]. The analysis found that this literature [2] was the primary source of heterogeneity. The intervention in this study was 25 mg empagliflozin with a treatment course of 12 weeks. Compared with other studies, there was no significant difference in the screening criteria of subjects and baseline data of population blood pressure in this literature. At the same time, the dose of SGLT2i was more prominent, and the treatment course was more protracted. Therefore, considering the combined effect of SGLT2i dose and course is the reason for the considerable heterogeneity, suggesting that the antihypertensive effect of the same type of SGLT2i may increase with the prolongation of the course. Keith [16, 30] leads to a similar conclusion: the effect of empagliflozin on blood pressure increased from 12 to 24 weeks, suggesting that at least 6 months of treatment is required for the full antihypertensive effect. In patients with type 2 diabetes combined with hypertension, aggressive medication to lower blood pressure is effective in reducing the incidence of stroke, heart failure and microangiopathy. The 2022 American Diabetes Association Standards of Medical Therapy for Diabetes recommend that patients with diabetes combined with hypertension who are at high cardiovascular risk may have their blood pressure reduced to < 130/80 mmHg as long as the target can be safely met. In these patients, symptomatic hypoglycaemic and antihypertensive therapy is the mainstay, with ACEI/ARB drugs recommended for blood pressure control. For those who need to combine multiple drugs to lower blood pressure, SGLT2i can be considered as the preferred option to reduce cardiac and renal damage while exerting the dual effect of lowering glucose and blood pressure and avoiding drug over-treatment.

Our study confirmed that SGLT2i could effectively reduce HbA1c in patients with type 2 diabetes mellitus and hypertension. Sensitivity analysis showed that this literature [17] was highly sensitive and was the primary source of heterogeneity. By analyzing the experimental design scheme of this literature, the dose of antihypertensive and antidiabetic drugs was not maintained at the pretreatment dose but changed at the investigator’s discretion, which impacted the final HbA1c and blood glucose level. In addition, the sample size of this study was small, the basic hypoglycemic regimen of patients was unknown, and the proportion of patients using DPP-4 inhibitor showed severe inclination (n > 50%). Therefore, the bias caused by the sample size and experimental protocol of the original hypoglycemic drug could not be excluded.

Controversial risk of serious adverse events (SAEs) such as genital infections, urinary tract infections (UTIs), and hypotension associated with SGLT2i has limited its clinical use. Younes [31] found a significantly higher risk of genital infections, urinary tract infections, and hypotension with SGLT2i compared to a placebo. This study preliminary confirmed that SGLT2i was well tolerated and did not increase the risk of hypoglycemia. Although the incidence of hypoglycemia was slightly higher in the dapagliflozin and placebo groups than in the placebo group, the difference was not statistically significant [12]. This study confirmed that SGLT2i does not increase the risk of urinary tract infection, the included literature is not heterogeneous, and the conclusion is reliable. Women are the main subjects of urinary tract infections, and there is no apparent relationship with whether they take empagliflozin [2]. A higher percentage of empagliflozin patients experienced genital infections, hypoglycemia events, and comparable rates of urinary tract infections compared with placebo [2]. There are consistent reports that SGL2i increases the risk of genital infection. Compared with metformin, SGLT2i has a similar glucose-lowering effect and additional cardiovascular protection but an increased potential risk of genital infections. Patients treated with Dapagliflozin had slight increases in volume depletion, urinary tract or genital infections, and renal impairment compared with placebo [15]. Michael [14] compared the incidence of adverse events between the dapagliflozin group and the placebo group and concluded that there was no statistical difference in the incidence of genital and urinary tract infections between the two groups. Our study concluded that SGLT2i had a 2.32-fold increased risk of genital infections compared with placebo, I^2^ = 16.9, RR = 2.32, 95% CI [1.57, 3.42], z = 4.23, p = 0.00, and the conclusion has a certain reference value and needs to be further confirmed by expanding the research sample. Sensitivity analysis suggested that this literature [9] was the primary source of heterogeneity. The risk of genital infection was significantly higher in an empagliflozin group than in a placebo group in two subgroups (refractory and non-refractory hypertension) in the literature (6.6% > 1.5%; 5.7% > 2.9%), but the document did not describe the baseline data of genital infection in different treatment groups in subgroups, so the possibility of uneven distribution of genital infection population at the time of grouping cannot be excluded. Compared with other literature, the sample size of subjects in this literature is more significant, which also causes heterogeneity to some extent. In subsequent studies, more multi-center clinical studies with rigorous designs and moderate sample sizes should be included for conclusion verification.

Although some studies have reported a higher frequency of serum creatinine increase or glomerular filtration rate (eGFR) decrease in the dapagliflozin group [15]. However, our study concluded that SGLT2i does not cause renal injury, which is consistent with the previously reported renal protective effect of SGLT2i [32]. SGLT2i also provided renoprotection by lowering intraglomerular hypertension by modulating the pre- and post-glomerular vascular tone. A meta-analysis confirmed that SGLT2i reduced the risk of progression of renal disease, and the magnitude of the benefit was positively correlated with baseline renal function, with more severe renal disease and weaker inhibition of the risk of disease progression.

Limitations of this study: (1) the included studies were few, and it was not possible to study the effect of SGLT2i on blood pressure control based on baseline blood pressure and the impact of cardiovascular risk factors. (2) The number of studies included was minimal, and the screening criteria for the literature were too wide, failing to analyze subgroups by geography, disease duration, gender, age, concomitant underlying condition, ethnicity, blood pressure, and underlying antihypertensive drug status. (3) The results of this study are somewhat biased due to the tiny study base, diverse location, ethnicity, and sample size. (4) The majority of research times ranged from 4 to 12 weeks, and SGLT2i’s long-term antihypertensive effectiveness is uncertain. (5) To further address these issues, additional clinical studies with sizable sample sizes, multicenter, prolonged study periods, and thorough study methods must be added.

There is a considerable risk of cardiovascular events in patients with type 2 diabetes mellitus and hypertension, and antihypertensive management strategies for this high-risk population are particularly important. In our study, the efficacy and safety of SGLT2i in patients with type 2 diabetes mellitus and hypertension were verified. SGLT2i has dual efficacy in lowering blood pressure and blood glucose and has a protective effect on cardiovascular, cerebrovascular, renal, and other composite outcomes and mortality. We can try to employ SGLT2i as an adjuvant medication in the first-line antihypertensive regimen for patients with type 2 diabetes and hypertension because this conclusion has some therapeutic work-guiding significance.

## Data Availability

The datasets used and/or analysed during the current study are available from the corresponding author on reasonable request. All data generated or analysed during this study are included in this published article and its Additional files.
